# Pharmacokinetics of Cannabidiol Following Intranasal, Intrarectal, and Oral Administration in Healthy Dogs

**DOI:** 10.3389/fvets.2022.899940

**Published:** 2022-06-07

**Authors:** Dakir Polidoro, Robin Temmerman, Mathias Devreese, Marios Charalambous, Luc Van Ham, Ine Cornelis, Bart J. G. Broeckx, Paul J. J. Mandigers, Andrea Fischer, Jan Storch, Sofie F. M. Bhatti

**Affiliations:** ^1^Small Animal Department, Small Animal Teaching Hospital, Faculty of Veterinary Medicine, Ghent University, Ghent, Belgium; ^2^Department of Pathobiology, Pharmacology and Zoological Medicine, Faculty of Veterinary Medicine, Ghent University, Ghent, Belgium; ^3^Clinic for Small Animals, Department of Neurology, University of Veterinary Medicine Hannover, Hannover, Germany; ^4^Laboratory of Animal Genetics, Faculty of Veterinary Medicine, Ghent University, Ghent, Belgium; ^5^Department of Clinical Sciences, Faculty of Veterinary Medicine, Utrecht University, Utrecht, Netherlands; ^6^Centre for Clinical Veterinary Medicine, Ludwig Maximilian University of Munich, Munich, Germany; ^7^CBDepot, Teplice, Czechia

**Keywords:** Canine, cannabinoid, *Cannabis sativa*, phytocannabinoid, tetrahydrocannabinol

## Abstract

The therapeutic potential of cannabidiol (CBD), a non-psychtropic component of the *Cannabis sativa* plant, is substantiated more and more. We aimed to determine the pharmacokinetic behavior of CBD after a single dose *via* intranasal (IN) and intrarectal (IR) administration in six healthy Beagle dogs age 3–8 years old, and compare to the oral administration route (PO). Standardized dosages applied for IN, IR and PO were 20, 100, and 100 mg, respectively. Each dog underwent the same protocol but received CBD through a different administration route. CBD plasma concentrations were determined by ultra-high performance liquid chromatography-tandem mass spectrometry before and at fixed time points after administration. Non-compartmental analysis was performed on the plasma concentration-time profiles. Plasma CBD concentrations after IR administration were below the limit of quantification. The mean area under the curve (AUC) after IN and PO CBD administration was 61 and 1,376 ng/mL*h, respectively. The maximal plasma CBD concentration (C_max_) after IN and PO CBD administration was 28 and 217 ng/mL reached after 0.5 and 3.5 h (T_max_), respectively. Significant differences between IN and PO administration were found in the T_max_ (*p* = 0.04). Higher AUC and C_max_ were achieved with 100 mg PO compared to 20 mg IN, but no significant differences were found when AUC (*p* = 0.09) and C_max_ (*p* = 0.44) were normalized to 1 mg dosages. IN administration of CBD resulted in faster absorption when compared to PO administration. However, PO remains the most favorable route for CBD delivery due to its more feasible administration. The IR administration route is not advised for clinical application.

## Introduction

Several lines of evidence have supported a therapeutic potential of cannabis derivatives, in particular phytocannabinoids, in human and veterinary medicine (1–7). Cannabidiol (CBD) and Δ9-tetrahydrocannabinol are the most abundant phytocannabinoids extracted from the *Cannabis sativa* plant (8–10), with CBD being the most promising since this molecule is devoid of the psychoactive side effects exhibited by Δ9-tetrahydrocannabinol (9, 11, 12).

Phytocannabinoids have a complex and variable pharmacokinetic and pharmacodynamic profile. They show a prominent hepatic first-pass effect and therefore have a low oral bioavailability (13). Their target, the endocannabinoid system, is composed primarily of CB1 receptors, expressed mainly by central and peripheral neurons, and CB2 receptors, expressed mainly by immune cells (14–16), suggesting a therapeutic value of CBD for numerous medical conditions in humans because of its potential neural (2, 17) and immunomodulatory properties (18). Therapeutic applications of CBD in humans include epilepsy (1, 19–21), Alzheimer's disease (22) and multiple sclerosis (23, 24). In the veterinary medicine, therapeutic applications of CBD in dogs include osteoarthritis-associated pain (6, 25, 26), aggressive behavior (27), and epilepsy (7, 28). CBD is generally administered orally, but its low bioavailability, which is estimated to be <10% in humans (19, 29), continues to be a main issue in clinical trials (30). In healthy dogs, it has been shown that administration of oral CBD is associated with a low bioavailability as well, ranging from 13 to 19%, most likely due to its first-pass phenomenon in the liver (31). The aforementioned limitations indicate the necessity to explore alternative delivery routes.

The purpose of this study was to determine the pharmacokinetic behavior of CBD after a single dose *via* intranasal (IN) and intrarectal (IR) administration in healthy Beagle dogs and compare this to the more widely used oral administration route (PO). The plasma CBD concentrations were evaluated over a period of 60 h post-administration. We hypothesized that CBD delivered *via* IN administration would avoid first-pass liver effect and CBD delivered *via* IR administration would partially avoid liver metabolization and therefore higher plasma concentrations and subsequent exposure would be achieved compared to the PO administration route.

## Materials and Methods

### Animals

Six neutered adult laboratory Beagle dogs (four females, two males), 3–8 years of age, weighing an average of 12 kg (range, 7.3–14.4 kg) were included in a randomized crossover study. A sample size of 6 was found to be the minimum sample size based on a power analysis with the following settings: the smallest relevant difference of 5 [with σ = 4, values based on (32)], based on a one-sample *t*-test exact solution with a non-central t-distribution, taking none-detection in one dog into account (31), with a power of ≥0.8 and at the α-level of 0.05. The study protocol was reviewed and approved by the Ethical Committee of the Faculties of Veterinary Medicine and Bioscience Engineering, Ghent University (EC 2018-42) and all manipulations were performed according to good animal practice. Care was taken to avoid stress and anxiety. No animals were sacrificed. The dogs were provided by the Small Animal Department of the Faculty of Veterinary Medicine and were purchased from Marshall BioResources (North Rose, New York, United States of America). The dogs were socially-housed in small groups (2 to 8 dogs), according to the European and Belgian legislation and received environmental enrichment (Directive 2010/63/EU, KB 29/05/2013). The bedding material in the inner part of the housing facility consisted of wood shavings. The dogs had permanent access to an outside area of 15 m^2^ and twice a day they were allowed to run and play outside in an enclosed play area, enriched with climbing platforms, hiding places and small bushes. In addition, the dogs were regularly walked by students of the Faculty of Veterinary Medicine. Food was withheld for at least 12 h before the start of the experiments, but water was provided *ad libitum*.

### Study Design

The dogs were randomly allocated to a 3-way crossover design by the principal investigator (DP), using an online randomization program (www.randomizer.org). Following a two-week wash-out period, each dog underwent the same protocol but received CBD through a different administration (IN, IR, PO) route. The first blood sample (T0) was taken 10 min before the CBD administration.

### CBD Administration and Sample Collection

For the IN administration, a polyethylene glycol (PEG):sodium chloride (NaCl) 0.9% (50:50) solution containing 20 mg of synthetic CBD (2-[(1R,6R)-3-methyl-6-prop-1-en-2-yl-1-cyclohex-2-enyl]-5-pentylbenzene-1,3-diol) per dog was given *via* a mucosal atomization device (MAD NasalTM, Wolfe Tory Medical, South Salt Lake City, Utah, United States). The total volume was fixed at 1 mL and was divided equally over the two nostrils. During the intranasal delivery, dogs were held in sternal recumbency with the head and neck gently dorsoflexed and were kept in this position for ~0.5 min after intranasal administration.

For the IR administration, dogs were first taken outside for a walk to avoid defecation during and after administration. Thereafter, a suppository containing 100 mg of CBD (Cannef^®^ synthesized CBD suppositories 100 mg, CB21 Pharma s.r.o., Prague, Czech Republic) was gently administered in each dog manually in the rectum.

For the oral administration, a tablet containing 100 mg of CBD (Cannamed^®^ synthesized CBD tablets 100 mg, xMed 21 s.r.o., Prague, Czech Republic) was administered together with a small amount (≅10 g) of highly digestible commercial canned food (Hill's^®^ Prescription Diet^®^ i/d^®^ Canine, Hill's Pet Nutrition Inc., Topeka, USA).

The tablets for oral administration and suppositories for rectal use (100 mg), according to the manufacturers' specification, were commercially available (www.CBDepot.eu), and the IN dose was a self-developed formulation using analytical standard dissoluted in PEG.

Blood samples (2 mL) were collected from the *vena jugularis* 10 min before CBD administration (T0) and at 15, 30, 45 min and 1, 1.5, 2, 3, 4, 6, 8, 10, 12, 24, 36, 48, and 60 h after all routes of CBD administration. Blood samples were immediately transferred into tubes containing ethylenediamine tetraacetic acid and the plasma was immediately separated by centrifugation at 3,500 rpm for 5 min at 2°C. The plasma was then stored at −80°C until analysis.

Adverse reactions during and after CBD administration were recorded. Attention was given to sneezing and reverse sneezing, coughing, head shaking, snorting and licking during IN administration, nausea, vomiting and salivation during PO administration and defecation after IR administration.

### Quantification of CBD in Plasma

#### Chemicals and Reagents

Ultrapure H_2_O was obtained *via* a Milli-Q water purification system (Merck Millipore, Overijse, Belgium). Standards of CBD (10 mg/mL in EtOH) and internal standard CBD-d3 (100 μg/mL) were purchased from Sigma-Aldrich (Overijse, Belgium). All solvents were of analytical grade. Acetonitrile was purchased from Thermo Fisher Scientific Inc. (Erembodegem, Belgium). Formic acid was obtained from VWR™ (Leuven, Belgium).

#### Sample Preparation

One hundred microliter of the plasma sample was spiked with 25 μL of internal standard (CBD-d3) solution (400 ng/mL) and vortex mixed (±15 s). Next, 275 μL of acetonitrile was added to the samples and again vortex mixed (±15 s). After mixing, the samples were centrifuged (13,000 rpm, 10 min). Thereafter, the liquid layer was transferred to an autosampler vial. Finally, an aliquot (5 μL for concentrations below 250 ng/mL, 1 μL for concentrations between 250 and 1,500 ng/mL) was injected into the ultra-high-performance liquid chromatography with tandem mass spectrometry (LC—MS/MS) system.

#### LC-MS/MS

Chromatographic separation was performed using an Acquity UPLC HSS-T3 column (100 × 2.1 mm, dp: 1.8 μm) in combination with a guard column of the same type (Waters NV/SA, Asse, Belgium). The gradient elution programme consisted of two mobile phases (A and B). Mobile phase A and B were 0.1% formic acid in ultrapure H_2_O and 0.1% formic acid in methanol, respectively. The following program was applied: 0 min (60% A, 40% B), 0–2 min (linear gradient to 100% B), 2–4 min (100% B), 4–4.1 min (linear gradient to 60% A, 40% B) and 4.1–8 min (60% A, 40% B). Flow rate was set at 0.4 mL/min. The LC eluent was interfaced to a Xevo TQ-XS triple quadrupole mass spectrometer (Waters NV/SA, Asse, Belgium) with ion source heated electrospray ionization operating in positive ionization mode. Acquisition was performed in selected reaction monitoring mode. For CBD and internal standard, the following two most intense product ions were followed: CBD: mass-to-charge ratio 315.08 > 193.00/122.96 and CBD-d3: mass-to-charge ratio 318.12 > 196.03/122.96. The LC-MS/MS analytical methods were validated using matrix-matched calibrator and quality control samples, based on blank plasma of untreated dogs. The limit of quantification (LOQ) was 1 ng/mL. The LC-MS/MS analyses were conducted in accordance with the international guidelines (33–35).

### Pharmacokinetic Analysis

Non-compartmental analysis was performed on the plasma concentration-time profiles using Phoenix 8.4 (Certara LP, NJ, USA). All dosing groups were included in the analysis, except for the IR administration data because of the low plasma concentrations (around LOQ). The following pharmacokinetic parameters were calculated: area under the plasma concentration-time curve, from 0 to infinity (AUC_0−inf_); maximal plasma concentration (C_max_) and time to maximal plasma concentration (T_max_); terminal elimination half-life; elimination rate constant and mean residence time. Total body clearance and volume of distribution after IN and PO administration were not corrected for their respective bioavailabilities. The relative bioavailability of the IN administration when compared with the commercial oral product was calculated according to the following formula:


$$\begin{array}{l} relative\;F=100\;\times \;\frac{{mean\;AUC\;IN\;}_{\left(0-inf\right)}\times \;Dose\;PO}{mean\;{AUC\;PO}_{\left(0-inf\right)}\;\times Dose\;IN} \end{array}$$

Due to the dose discrepancy between the PO and IR administration (100 mg) and the IN administration (20 mg) and to facilitate comparison of the systemic exposure between the administration routes, the AUC and C_max_ of PO/IR and IN were normalized for dose by dividing by 100 and 20, respectively.

### Statistical Analysis

The statistical analysis was conducted in R version 4.0.2 (“Taking off again”). Significance was set at α ≤ 0.05. A Wilcoxon signed rank test was used to compare the IN and PO administration routes.

## Results

**Figure 1:**
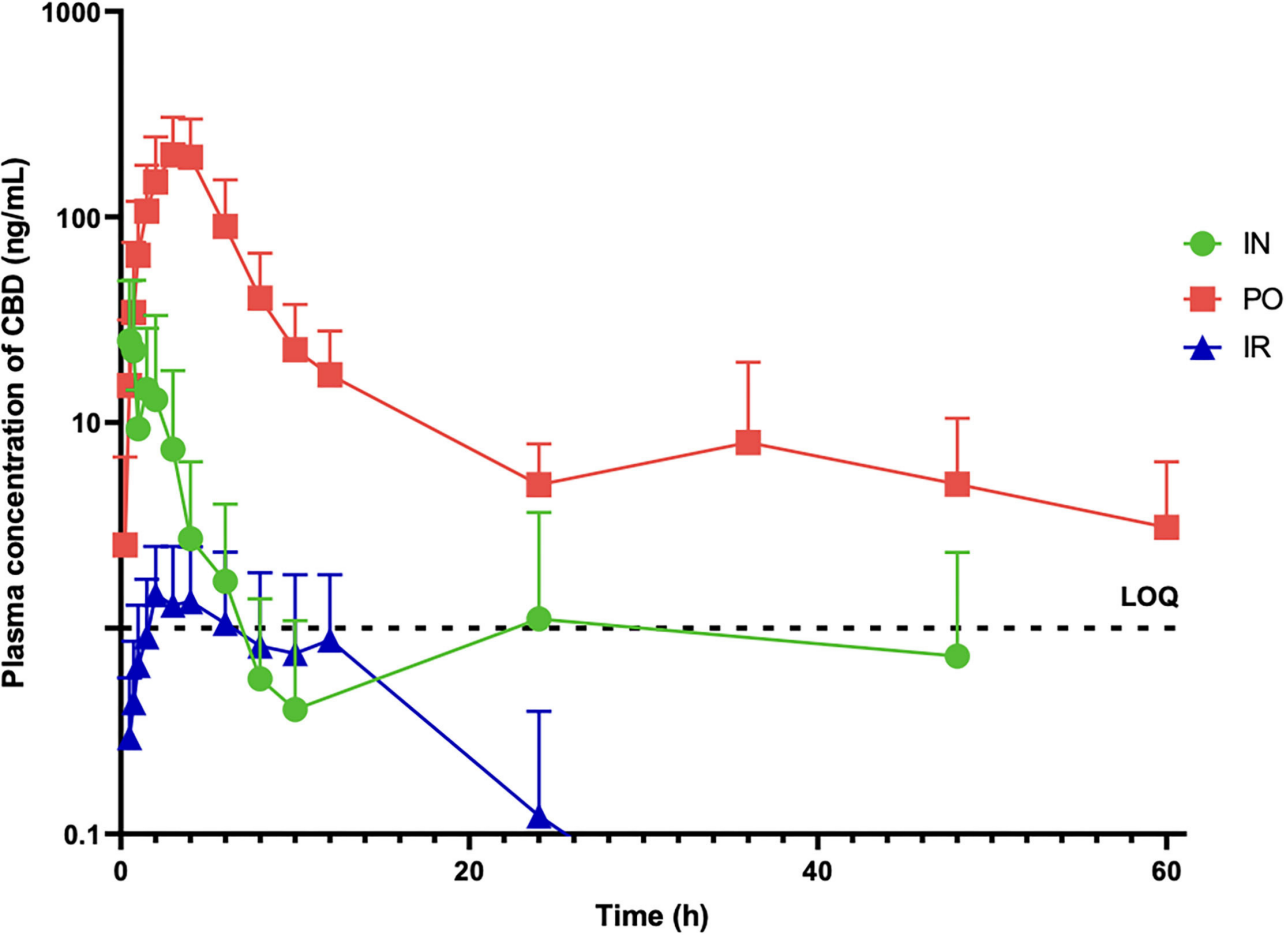
Mean ± SD plasma concentrations of CBD (ng/mL) in six dose administered a single dose of CBD intranasally (20 mg) (IN), orally (100 mg) (PO) and intrarectally (100 mg) (IR).

Mean ± SD plasma CBD concentrations after PO, IN and IR administration were determined at 17 time points over 60 h post administration and are displayed in Figure 1. The mean AUC_0−inf_ after IN and PO CBD administration before dose normalization was 61.31 and 1376.03 ng/mL^*^h and after normalization to 1 mg dosages was 3.06 and 13.76 ng/mL^*^h, respectively. The maximal plasma CBD concentration (C_max_) after IN and PO CBD administration before dose normalization was 27.96 and 216.76 ng/ml and 1.39 and 2.16 ng/mL after dose normalization, reached at 0.5 and 3.5 h (T_max_), respectively. Significant differences between IN and PO administration routes were found in the T_max_ (*p* = 0.04) but no significant differences were found in the AUC normalized for dose (*p* = 0.09) and C_max_ normalized for dose (*p* = 0.44). The different pharmacokinetic parameters after PO and IN administration are shown in Table 1.

**Table 1:** Pharmacokinetic parameters (mean ± SD) of CBD in 6 dogs administered a single dose of CBD intranasally (20 mg) (IN) and orally (100 mg) (PO).

**Pharmacokinetic parameter**	**IN**	**PO**
AUC_(0−∞)_ (ng/mL^*^h)^*^	61.31 ± 88.22	1376.03 ± 828.95
C_max_ (ng/mL)^*^	27.96 ± 25.29	216.76 ± 108.51
AUC_(0−∞)_ (ng/mL^*^h)^**^	3.06 ± 4.41	13.76 ± 8.28
C_max_ (ng/mL)^**^	1.39 ± 1.26	2.16 ± 1.08
T_max_ (h)^†^	0.49 ± 0.29	3.50 ± 0.55
T_1/2el_ (h)	7.02 ± 7.97	15.65 ± 2.82
k_el_ (1/h)	0.52 ± 0.54	0.045 ± 0.007
MRT (h)	10.30 ± 14.04	13.07 ± 3.61
Relative F (%)	22.28%	/

*AUC_(0−∞)_, area under the plasma concentration-time curve from 0 to infinity post-administration; C_max_, maximal plasma concentration; T_max_, time to maximal plasma concentration; T_1/2el_, terminal elimination half-life; k_el_, elimination rate constant; MRT, mean residence time; F, absolute bioavailability*.

* *Doses for AUC_(0−∞)_ and C_max_ before dose normalization*.

** *Doses for AUC_(0−∞)_ and C_max_ after dose normalization to 1 mg CBD*.

† *Significant differences between administration routes*.

Due to the very low (below LOQ) plasma CBD concentrations (Figure 1) obtained after IR administration, no pharmacokinetic analysis was conducted for this administration route.

Two dogs showed sneezing and 1 dog showed head shaking after IN administration. These symptoms were seen immediately after IN administration and disappeared over a period of 30 s. All 6 dogs showed good compliance with PO administration, where the tablets were spontaneously ingested together with the small amount of highly digestible food. The IR administration of the CBD suppository went uneventful. No signs of nausea, vomiting or salivation were seen during and after PO administration and no defecation was observed during and after the IR administration.

## Discussion

To the best of our knowledge, this is the first study examining the pharmacokinetic profile of CBD in healthy dogs after IN and IR administration. The pharmacokinetic parameters were compared with the more widely used oral route of administration. The plasma CBD concentrations after IR administration were below the LOQ and were therefore not used in our analysis. We hypothesized that CBD delivered *via* IN administration would bypass liver metabolization largely and *via* IR administration partially and therefore higher plasma concentrations would be achieved, in comparison with the PO administration route. However, this hypothesis was not confirmed, since the oral administration route still showed a numerically higher mean exposure and maximal concentration normalized for dose when compared to the IN and IR administration routes albeit not statistically significant.

IN drug delivery is non-invasive, pain-free and easy. The mucosal atomization device converts the liquid drug into a fine mist and is used to deliver it into the nasal cavity consequently reaching the nasal mucosa. The nasal mucosa provides a large, particularly vascular absorptive surface adjacent to the brain and offers a direct pathway for drug absorption into the bloodstream, avoiding the first-pass hepatic phenomenon (36–39). As the IN route, the oromucosal route is also an easy and pain-free drug delivery method able to circumvents some of the problems associated with the PO route, such as avoidance of first-pass hepatic metabolism (40). The oromucosal route can also provide a rapid onset of action (41), as long as the exposure times to the oral mucosal lining are adequate and a method of preventing washout of the drug by saliva is present (42), which is practically not possible in awake dogs due to lack of compliance (40). In our study, the mean T_max_ after IN administration was reached significantly faster (0.49 h) compared with the PO administration (3.5 h), but on the other hand, there was no significant difference between the mean AUC normalized for dose and mean C_max_ normalized for dose when comparing IN and PO administrations. It is worth to mention that a substantial proportion of the oromucosal delivered dose of CBD may actually be absorbed through the gastrointestinal tract (43) and this phenomenon may also be seen with IN drug administration (44), which could have influenced the velocity of the IN T_max_ concentrations in this study. Although drug delivery *via* IN administration is quickly absorbed and bypasses first-pass effect, other factors may have influenced IN CBD plasma concentrations in our study, such as the solvent formulation (PEG) used and the high lipophilicity of CBD. It is believed that PEG-only formulations are associated with a higher CBD permeation (45). In our study, CBD was intranasally delivered in a PEG:NaCl 0.9% (50:50) solution. The viscosity of a PEG-only formulation would be too high and would not turn into a fine mist of particles when administered *via* the mucosal atomization device. Paudel et al. (45) evaluated the pharmacokinetic parameters after IN CBD administration *via* a surgical procedure in anesthetized rats, using different solvent formulations containing PEG. Rats that received IN CBD with PEG alone, showed a 3.5-fold increase in mean AUC when compared to the group of rats that received IN CBD containing 50% of PEG, 35% saline and 15% ethanol in the solvent solution. Another explanation for the lower IN absorption could be the extreme lipophilicity of CBD (Log *P* 6.3) (46), which may make crossing the aqueous media of the nasal mucosa and other polar secretions difficult (45). Furthermore, the dogs in our study were not sedated nor anesthetized, which might have facilitated nasal drug delivery and possibly increased the AUC as well. Vlerick et al. (39) achieved a complete bioavailability when ketamine was administered intranasally in sedated dogs and this was associated with a lower risk of spilling and swallowing of the drug. Two dogs in our study sneezed and one dog was head shaking after the IN administration, which could have led to partial spilling of the drug out of the nasal cavity. On the other hand, we believe that not anesthetizing or sedating the dogs would avoid any possible drug-drug interaction that could consequently affect CBD pharmacokinetics and would not reflect the in-practice situation. The interaction between cannabinoids and volatile and intravenous anesthetic agents is equivocal, with evidence limited to animal studies, case reports and limited human studies (47). Also, it might have been possible that CBD concentrations after IN delivery were higher in the cerebrospinal fluid compared to plasma, but this was not analyzed in our study.

Main advantages of the oral administration of CBD include standardized concentrations and doses, and an easy administration route, where oils and capsules currently allow for more convenient and accurate dosing in comparison with other oral formulations (48). Besides the CBD oil, other oral formulations for dogs can be acquired, including soft chews, soft gel capsules and tablets (49). In general, CBD in oil suspensions designed for oral and oromucosal administration are currently favored and appears to be the preferred method of delivery for absorption (7, 40, 50). Pharmacokinetic analysis demonstrated that the CBD-infused oil formulation resulted in higher C_max_ and AUC than oral microencapsulated oil beads and CBD-infused transdermal cream (50). Small volumes of CBD oil might slowly transcend the esophagus into the stomach, which could possibly prolong its absorption, but on the other hand, CBD in a soft chew presentation is more likely to create a food bolus that it is delivered quickly to the stomach, resulting in a quicker digestion and absorption (51). Soft chews are currently the most popular dosage-form treats available in the marketplace for dogs (52). CBD has a high lipophilicity and its administration in a lipid solvent, such as medium-chain triglycerides oil for example, may increase the bioavailability of CBD (53). In a study in rats, the administration of oral CBD together with lipid compounds increased the bioavailability of CBD by almost 3 times when compared to non-lipid formulations (54). Oral dosing with CBD in an oil base may enhance absorption, but may enhance further by inclusion in a food matrix (51). Drugs with a high lipophilicity and that are administered orally in a lipid solution can precipitate in the gastrointestinal tract, resulting in an absorption rate slower than elimination (55). In a human study, the administration of CBD together with a high-fat caloric meal is used as a potential method to increase the oral bioavailability of CBD (43). This method has also been used in dogs receiving oral cannabinoid, where they were fed at the time of administration to promote cannabinoid absorption (56).

Higher plasma concentrations and exposure were achieved for practically applicable oral dosages than for applicable IN dosages for the examined products. The PO CBD dose in our study ranged from 7 to 13 mg/kg. Deabold et al. (51) was able to obtain similar concentrations when determining single-dose oral pharmacokinetics of CBD in healthy dogs using a lower dose (2 mg/kg), achieving a mean C_max_ of 301 ng/mL at 1.4 h (T_max_) and a mean AUC of 1,297 ng-h/mL. This is possibly due to the use of an infused soft chew treat made with a glycerol/starch/fiber base which should be easily digestible and appears to deliver approximately two and a half times the concentration. As in our study, other research groups also used a higher oral dose in dogs (10 mg/kg) and a mean C_max_ was reached, between 578 and 1,868 ng/mL, respectively (50, 57). The mean AUC found in our study after PO administration was 1,376 ng/mL^*^h. Bartner et al. (50) showed a mean AUC between 8,820 ng/mL^*^h (10 mg/kg of CBD-infused oil) and 6,180 ng/mL^*^h (10 mg/kg of microencapsulated oil) in healthy dogs. CBD has a high affinity for lipids and low water solubility (58), and consistent with its lipophilicity CBD administered orally was not detected in 50% of the dogs, in which CBD was administered as a powder within a gelatin capsule (31). Therefore, if given orally, it is best absorbed in the presence of fat, oils or polar solvents (59). In our study, oral CBD was administered within a tablet with a small amount of highly digestible wet food containing 15% of fat (as previously mentioned) and this could explain why we observed a lower CBD exposure compared to studies using oil-based CBD (50) but still a better absorption when comparing our results to other studies (31).

The rectum offers a practical delivery route for several drugs and is a relatively easy and quick method when oral administration is not feasible or when intravenous access is not available. In one study with healthy Beagle dogs, THC administered rectally with suppositories in a lipophilic base (Witepsol H15), had a bioavailability of ~67% (60). Intestinal absorption and bioavailability depend on several factors such as drug solubility in the gastro-intestinal environment, permeability of the drug through the enterocyte membrane, activity of efflux transporters and metabolizing enzymes (61). In our study, plasma CBD concentrations after IR administration were extremely low and therefore the pharmacokinetic parameters were not analyzed. The suppositories used in our study contained a formulation of glycerol monostearate, which is a more hydrophilic substance (Log *P* 7.4) (62). The use of suppository formulations in lipophilic bases was previously associated with a higher absorption and bioavailability of cannabinoids (60), thus use of more lipophilic bases could have increased plasma CBD concentrations after IR administration in our study. Overall, IR administration of CBD under the currently used formulation is not advised due to its inconsistent and low plasma concentrations.

The most frequently observed adverse effect associated with the IN route was short sneezing in two dogs and head shaking in one dog. Sneezing or snorting reaction and head shaking during or after intranasal administration have also been reported in three other studies, where dogs received IN diazepam (44), IN midazolam (38, 63) and IN ketamine (39). In our study, a PEG:NaCl 0.9% (50:50) solution was used as a solvent for the IN CBD administration. In humans, PEG has been reported to induce mild local toxicity to the nasal mucosa (64, 65), which could induce local irritation and a displeasing sensitivity. Nevertheless, all three administration routes were easy to perform and generally well-tolerated by all dogs.

The major limitation of this study was the lack of an IV route group, which could have provided some more consistent pharmacokinetic data. There are also potential limitations regarding the IN route, including spilling of the drug due to sneezing and swallowing of a part of the dose administered.

## Conclusion

The IN, IR and PO single administration of 20, 100, and 100 mg CBD, respectively, was well-tolerated by all of the dogs. PO remains the most favorable route for CBD delivery due to its more feasible administration. Nevertheless, IN administration of CBD provided a faster blood absorption when compared to the PO and IR CBD administration. These findings encourage the use of IN CBD in veterinary medicine as a possible alternative when PO route is not possible. The IR administration route is not advised for clinical application.

## Data Availability Statement

The original contributions presented in the study are included in the article, further inquiries can be directed to the corresponding author/s.

## Ethics Statement

The animal study was reviewed and approved by Ethical Committee of the Faculties of Veterinary Medicine and Bioscience Engineering, Ghent University (EC 2018-42).

## Author Contributions

DP and SB were responsible for conception of the study, writing–original draft, and approval of the submitted manuscript. RT and MD were responsible for conception of the study, acquisition of the data, data analysis and interpretation, pharmacokinetic evaluation, and writing–review and editing of the manuscript. BB was responsible for statistical analysis and writing–review and editing of the manuscript. MC, LH, IC, PM, AF, and JS were responsible for writing–review and editing of the manuscript. All authors contributed to the article and approved the submitted version.

## Conflict of Interest

JS was employed by company CBDepot. The remaining authors declare that the research was conducted in the absence of any commercial or financial relationships that could be construed as a potential conflict of interest.

## Publisher's Note

All claims expressed in this article are solely those of the authors and do not necessarily represent those of their affiliated organizations, or those of the publisher, the editors and the reviewers. Any product that may be evaluated in this article, or claim that may be made by its manufacturer, is not guaranteed or endorsed by the publisher.

## Abbreviations

AUC: area under the curve
CBD: cannabidiol
C_max_: maximal plasma concentration
IN: intranasal
IR: intrarectal
LC-MS/MS: liquid chromatography with tandem mass spectrometry
LOQ: limit of quantification
NaCl: natrium chloride
PEG: polyethylene glycol
PO: oral
T_max_: time to maximal plasma concentration.
